# Reversible central adrenal insufficiency in survivors of COVID-19: results from a 24-month longitudinal study

**DOI:** 10.1530/EC-24-0086

**Published:** 2024-08-12

**Authors:** Saroj Kumar Sahoo, Jayakrishnan C Menon, Nidhi Tripathy, Monalisa Nayak, Subhash Yadav

**Affiliations:** 1 Department of Endocrinology, Sanjay Gandhi Postgraduate Institute of Medical Sciences, Lucknow, India; 2 Division of Endocrinology, Mid and South Essex NHS Trust, Broomfield, UK; 3 Department of Liver Intensive Care Unit, King’s College Hospital, London, UK

**Keywords:** COVID-19, SARS-CoV-2, hypothalamic–pituitary–adrenal axis, adrenal insufficiency

## Abstract

**Objective:**

We studied the temporal course of hypothalamic–pituitary–adrenal (HPA) dysfunction in patients with coronavirus disease 2019 (COVID-19).

**Methods:**

Three hundred and two patients (median age 54 years (interquartile range (IQR) 42–64), 76% males) were recruited. The HPA axis was evaluated by morning cortisol and adrenocorticotrophic hormone (ACTH) at admission (*n* = 232). Adrenal insufficiency (AI) during acute illness was defined using a morning cortisol <83 nmol/L. AI at 12 months follow-up was defined using a peak cortisol <406 nmol/L in the ACTH stimulation test (APST) (*n* = 90). Those with AI at 12 months were further assessed by APST every 6 months for recovery of hypoadrenalism.

**Results:**

The median morning cortisol and ACTH levels during COVID-19 were 295 (IQR 133–460) nmol/L and 3.9 (0.8–6.9) pmol/L, respectively. AI was present in 33 (14%) patients; ACTH was elevated in three and low or inappropriately normal in the rest 30 patients. At 12 months, AI was seen in 13% (12/90) patients, with all cases being hypothalamic–pituitary in origin; five (42%) of them had not met the diagnostic criteria for AI during COVID-19. AI diagnosed at admission persisted at 12 months in seven patients and recovered in seven; the remaining 19 patients were lost to follow-up. The presence of AI at 12 months was independent of severity and steroid use during COVID-19. A morning cortisol <138 nmol/L during COVID-19 predicted the presence of AI at 12 months. All patients showed recovery of the HPA axis in the ensuing 12 months.

**Conclusion:**

Central AI was common during acute COVID-19 and at 12 months of follow-up. AI can be late onset, developing after recovery from COVID-19, and was transient in nature.

## Introduction

Coronavirus disease 2019 (COVID19) is a multisystem disease caused by severe acute respiratory syndrome coronavirus-2 (SARS-CoV-2) and has been a major public health challenge across the globe since its emergence in late 2019. The SARS-CoV-2 requires angiotensin-converting enzyme 2 (ACE2) receptor and transmembrane serine protease 2 (TMPRSS2) for gaining entry inside the cells (1). In humans, ACE2 and TMPRSS2 mRNAs are expressed in several endocrine glands, including the hypothalamus, pituitary, and adrenal cortex (2). Thus, it is possible that patients with COVID-19 may have hypothalamic–pituitary–adrenal (HPA) axis dysfunction both during the acute COVID-19 and/or following recovery from COVID-19.

Limited data on the prevalence of hypocortisolism in patients with acute COVID-19 provide contrasting results. In the largest study, hypocortisolism was not observed in 403 patients with COVID-19 (3). In contrast, other small-sized studies showed a variable prevalence of hypocortisolism ranging between 14% and 64% in patients with acute COVID-19 (4, 5, 6). The variable prevalence in these studies might be explained by smaller sample sizes and different cut-offs of morning cortisol used to define hypocortisolism.

Similarly, the limited literature on the prevalence of adrenal insufficiency (AI) among survivors of COVID-19 shows conflicting results. In the first one, a standard-dose 250 μg short Synacthen (tetracosactide acetate) test (SD-SST) failed to detect the presence of AI in 70 patients (7). In contrast, two other studies revealed AI in 13–16% of survivors but using a low-dose 1 μg Synacthen test (LD-SST) (8, 9). These studies were conducted after 3 months of recovery from acute COVID-19 and might have suffered from a few limitations. Although AI may develop soon after recovery from a critical illness (10), evaluation of HPA axis using SD-SST at this early stage may fail to detect cases of secondary AI as adrenals may retain sensitivity to adrenocorticotrophic hormone (ACTH) (11). The LD-SST has an advantage at this early stage of secondary AI, but it may suffer from performance error and resultant false-negative test (11, 12, 13). Glucocorticoids have been the mainstay of treatment of severe COVID-19, and short courses of high-dose glucocorticoids may even suppress the HPA axis for more than 2 weeks (14). Thus, it may be challenging to differentiate AI secondary to COVID-19 from glucocorticoid-induced AI in the early months after recovery from COVID-19. Hence, assessment at a further delayed period using SD-SST will provide a more accurate reflection of the HPA axis. Further, the temporal course of adrenal function is also not studied in patients with COVID-19. Hence, we evaluated the HPA axis both during COVID-19 and at 12 months after recovery to assess the persistence of AI and screen patients for delayed onset of AI. Those with AI were assessed every 6 months for recovery of the HPA axis.

## Materials and methods

### Participants

This was a single-centre prospective study carried out between July 2020 and June 2023 at a tertiary care public health institute in North India. Patients >18 years, admitted to our hospital with a diagnosis of COVID-19 between June 2020 and June 2021, were included in the study. COVID-19 was diagnosed based on RT-PCR positivity for SARS-CoV-2 in nasopharyngeal and/or throat swab samples. Patients receiving glucocorticoids or opiates prior to sampling, those who were pregnant, and those who had pre-existing pituitary or adrenal disease were excluded from the study. Those who were using medications affecting cortisol-binding globulin (estrogens) were also excluded from the study. Three hundred and two patients (median age 54 years, interquartile range (IQR) 42–64 years, 76% males) were recruited in this period. The study was done in compliance with Declaration of Helsinki. Written informed consent was obtained from all study participants or from the family members, if the patients were critically ill. The study was approved by the Ethics Committee of Sanjay Gandhi Postgraduate Institute of Medical Sciences (study number 2020-135-EMP-EXP-20).

**Figure 1:**
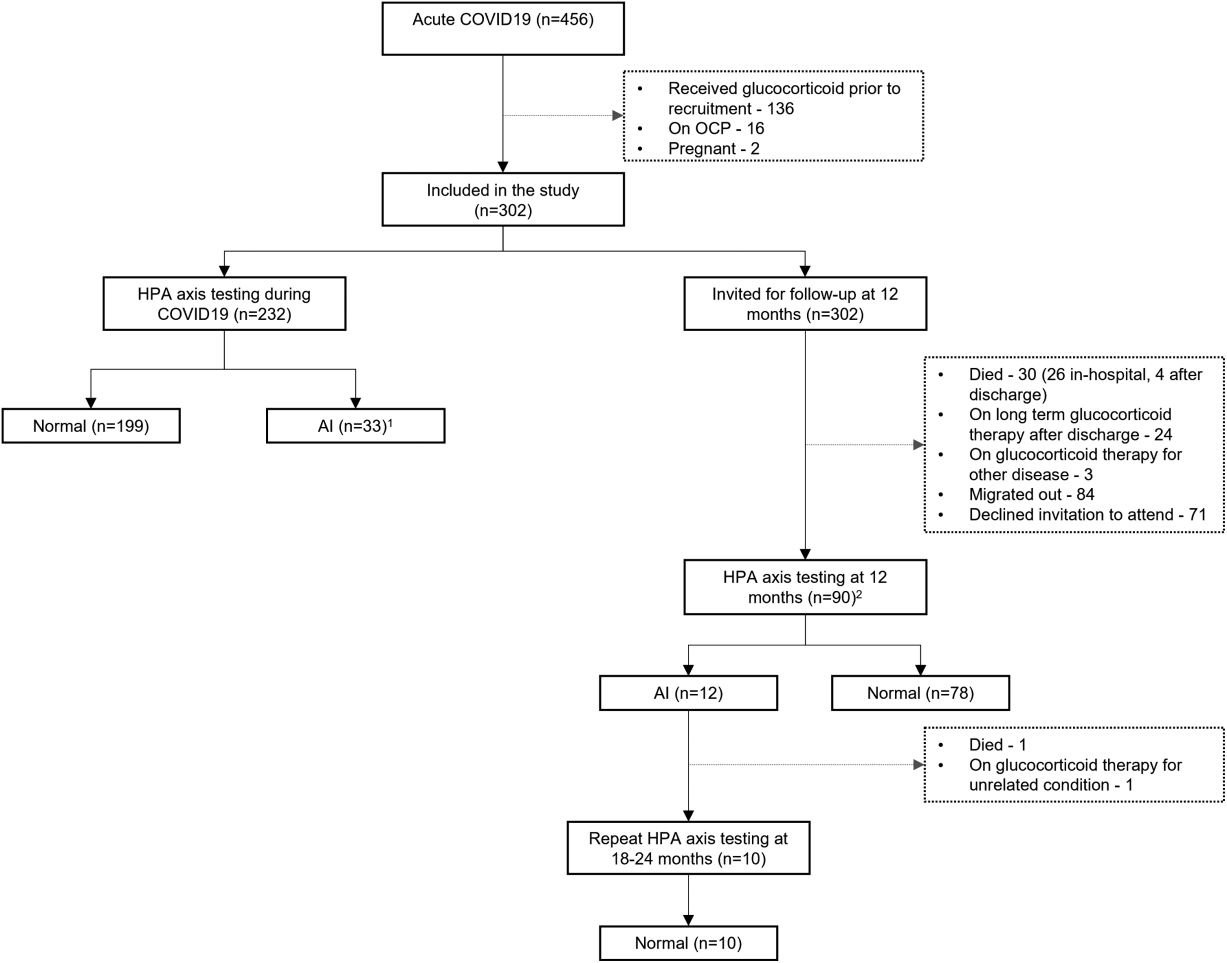
Flowchart showing recruitment of participants and their follow-up in the study. ^1^Of the 33 patients with AI during COVID-19, only 14 could turn up at 12 months for follow-up, and rest 19 were lost to follow-up. Of the 14 who were tested for the HPA axis at 12 months, AI persisted in seven, while it recovered in the rest. ^2^The 90 patients who were followed at 12 months included 35 patients who did not have a baseline cortisol measured during COVID-19. AI, adrenal insufficiency; COVID-19, coronavirus disease-2019; HPA, hypothalamic pituitary adrenal.

Of the 302 patients recruited for the study, baseline samples for morning cortisol and ACTH were available for 232 patients. For the assessment of the HPA axis at 12 months, we not only invited these 232 patients but also the remaining 70 patients without a baseline cortisol measurement (Fig. 1).

### Evaluation during acute COVID-19

All patients were assessed using a predesigned proforma for their clinical, biochemical, and radiological parameters. A venous blood sample was drawn between 08∶00 h and 09∶00 h within the first 48 h of the hospitalisation and was analysed for complete blood count, erythrocyte sedimentation rate, high-sensitivity C-reactive protein, procalcitonin, lactate dehydrogenase, ferritin, d-dimer, fibrin-degradation product, and fibrinogen on the same day. Baseline morning plasma and serum samples were also stored at −80°C until analysed for total cortisol, ACTH, free thyroxine (FT4), triiodothyronine (T3), thyroid-stimulating hormone (TSH), and the cytokines interleukin-6 (IL-6) and interleukin-8 (IL-8).

### Follow-up evaluation at 12–24 months

Of the 302 patients recruited, 30 (10%) died during the hospital stay or soon after the discharge. The remaining patients were invited for a follow-up 12 months after discharge from the hospital. The participants who had received steroids any time after the discharge were further excluded from the follow-up visits. In the final analysis, 90 patients were available for follow-up at 12 months (Fig. 1). They were assessed for clinical manifestations of long-COVID syndrome, which was defined as the continuation of or development of new symptoms 3 months after the initial SARS-CoV-2 infection, with these symptoms lasting for at least 2 months, with no other explanation (15). Blood samples were obtained between 08∶00 h and 09∶00 h for measurement of serum cortisol, ACTH, and dehydroepiandrosterone sulphate (DHEAS). This was followed by an intramuscular injection of ACTH (Acton Prolongatum®, Ferring Pharmaceuticals, Saint Prex, Switzerland) and sampling at 60 and 120 min for the assay of serum cortisol (Acton Prolongatum stimulation test, APST). A previous study on SARS had showed recovery of the HPA axis in the follow-up (16). Those who had AI further underwent APST at 6-month intervals looking for recovery of the HPA axis.

### Acton Prolongatum stimulation test (APST)

Current guidelines suggest the use of the SD-SST for the diagnosis of both primary and secondary AI. Synacthen or tetracosactide is a synthetic ACTH composed of first 24 amino acids of the ACTH(1–39) peptide. Synacthen is not marketed in many countries, including India. Acton Prolongatum^®^, widely available in India, is a synthetic porcine sequence corticotrophin composed of the 1–39 peptide reversibly bound to carboxymethylcellulose. The latter protects the peptide against enzymatic breakdown, thus prolonging its action. A dose of 25 IU shows a response in cortisol rise equivalent to a dose of 250 μg synacthen (17, 18). The diagnosis of AI using APST has been previously validated against ITT and SST (17, 18, 19).

### Assays

Serum total cortisol was assessed by electrochemiluminescence method (Elecsys Cortisol II assay, Roche Diagnostics). Serum FT4 (reference range 12–22 pmol/L), T3 (reference range 1.3–3.1 nmol/L), TSH (reference range 0.4–4 mIU/L), DHEAS (reference range: male 2.4–13.4 µmol/L, female (5–24 years) 1.8–11.0 µmol/L, female (25–45 years) 1.7–9.2 µmol/L, female (>45 years) 0.5–6.9 µmol/L) and plasma ACTH (reference range 1.6–13.9 pmol/L) were also assessed by the electrochemiluminescence method (Roche Diagnostics). IL-6 and IL-8 were analysed by cytometric bead array assay (BD Life Sciences, San Jose, CA, USA).

### Definitions

Severity of COVID-19 was defined by the WHO criteria as mild–moderate, severe, and critical illness (20). There is no consensus on diagnostic criteria for AI in acutely ill patients; hence, we used the standard cut-off used to define AI during acute COVID-19 (21, 22). During acute COVID-19, we did not use the peak stimulated cortisol in the APST for defining AI, as adrenal glands retain sensitivity to ACTH in the early stages of central AI and this may result in a false-negative result. AI during acute illness was defined as a morning cortisol <83 nmol/L (3 µg/dL) (21, 22). AI at 12 months was defined as a peak cortisol value <406 nmol/L (14.6 µg/dL) in the APST (23). We used a lower cut-off for peak cortisol, based on validation in the newer assays using monoclonal antibodies (23). These values were quite similar to the 2.5th percentile cut-off values for basal (104 nmol/L) and peak (396 nmol/L) cortisol, as evaluated in a local cohort of 24 healthy controls.

### Statistical analysis

Statistical analysis was performed using the Statistical Package for Social Sciences (SPSS) version 23 (IBM). Data were expressed as mean (s.d.), median (IQR), and percentage as appropriate. Continuous variables (all nonparametric) were compared using the Mann–Whitney test. The chi-square test/Fisher’s exact test was used to compare the proportions between groups. Pearson’s correlation was performed to test the correlation between variables. Binary logistic regression was performed to determine factors associated with mortality and the development of AI at follow-up. Factors that showed a significant correlation (*P* ≤ 0.05) with the response variable in the univariate model and those with biological significance were included in the final model; those which showed collinearity were excluded from the model. A receiver operating characteristic (ROC) analysis was performed to determine the appropriate cut-off of serum morning cortisol during acute infection that could predict the development of AI at follow-up. A *P*-value <0.05 was considered significant.

## Results

### Baseline evaluation during acute COVID-19 phase

The baseline characteristics can be found in Tables 1 and 2. Ninety- seven (32%) patients had severe or critical illness. Almost a third (108, 36%) required treatment with steroids for severe COVID-19. Twenty-six (9%) patients died during the hospital stay and another four died soon after discharge.

**Table 1:** Clinical characteristics of patients with and without adrenal insufficiency during acute COVID-19.

Parameter	Adrenal insufficiency, *n* = 33	Normal HPA axis, *n* = 199	*P*
Age (years)	56 (47–65)	55 (43–65)	0.44
Male gender (%)	26 (79)	143 (72)	0.43
Comorbidities (%)			
Diabetes mellitus	16 (49)	96 (49)	1.00
Hypertension	11 (33)	96 (49)	0.11
Obstructive airway disease	4 (12)	10 (5)	0.12
ASCVD	2 (6)	7 (4)	0.49
Chronic kidney disease	1 (3)	25 (13)	0.11
Presenting symptoms (%)			
Fever	22 (67)	139 (70)	0.68
Cough	19 (58)	90 (46)	0.21
Dyspnea	10 (30)	63 (32)	0.86
Anosmia	5 (15)	31 (16)	0.93
Myalgia	9 (27)	39 (20)	0.32
Vomiting	4 (12)	17 (9)	0.51
Diarrhea	3 (9)	10 (5)	0.35
Symptom duration (days)	5 (4–10)	5 (3–8)	0.44
ARDS (%)	7 (22)	28 (16)	0.37
Severe/critical disease (%)	11 (33)	71 (36)	0.78
Radiological evidence of pneumonia (%)	24 (73)	124 (66)	0.42
Supplemental oxygen (%)	7 (22)	51 (27)	0.58
Non-invasive ventilation (%)	4 (12)	18 (9)	0.60
Invasive ventilation (%)	2 (6)	11 (6)	0.93
Glucocorticoids (%)	14 (42)	62 (31)	0.21
Death (%)	3 (9)	21 (11)	0.79

Data are presented as median (interquartile range) and percentage as appropriate.

ARDS, acute respiratory distress syndrome; ASCVD, atherosclerotic cardiovascular disease; COVID-19, coronavirus disease-2019; HPA, hypothalamic–pituitary–adrenal.

**Table 2:** Laboratory parameters in patients with and without adrenal insufficiency during acute COVID-19.

Parameter	Adrenal insufficiency, *n* = 33	Normal HPA axis, *n* = 199	*P*
Hemoglobin (g/dL)	11.6 (10.4–13.1)	11.4 (9.8–13.1)	0.61
Total leukocyte count (cells/mm^3^)	10,050 (8205–17,450)	9180 (6325–14,000)	0.19
ESR (mm/h)	57 (11–75)	60.5 (18–84.3)	0.29
S. C-reactive protein (mg/dL)	29 (5.1–90.6)	41.5 (8.7–109.4)	0.26
S. Procalcitonin (ng/mL)	0.05 (0.03–0.17)	0.09 (0.03–0.34)	0.21
P. Fibrinogen (mg/dL)	514 (439–611)	522 (421–621)	0.72
P. d-dimer (ng/mL)	0.64 (0.28–2.03)	0.57 (0.18–2.00)	0.30
S. Lactose dehydrogenase (U/L)	388 (291–566)	438 (296–668)	0.55
S. Ferritin (µg/L)	390 (149.8–957)	418 (205–1152)	0.44
P. Interleukin 6 (pg/mL)	6.7 (5.0–117.0)	10.3 (5.0–42.2)	0.96
P. Interleukin 8 (pg/mL)	20.1 (5.2–76.3)	16.2 (8.1–52.5)	0.83
S. Morning cortisol, morning (nmol/L)	47.1 (32.1–65.8)	330.1 (201.9–488.7)	0.00
P. ACTH (pmol/L)	3.4 (0.6–6.6)	3.9 (0.8–7.1)	0.42
S. Free thyroxine (pmol/L)	16.8 (14.7–19.2)	17.2 (14.9–19.5)	0.61
S. Triiodothyronine (nmol/L)	1.6 (1.2–1.9)	1.5 (1.3–1.9)	0.83
S. Thyroid-stimulating hormone (mIU/L)	1.5 (0.8–2.7)	1.9 (1.1–3.2)	0.23

Data are presented as median (interquartile range) and percentage as appropriate.

ACTH, adrenocorticotrophic hormone, COVID-19, coronavirus disease 2019, ESR, erythrocyte sedimentation rate; HPA, hypothalamic–pituitary–adrenal; P., plasma; S., serum.

Baseline cortisol measurements were available for 232 patients admitted during COVID-19. The median morning cortisol and ACTH levels during the acute illness were 295 (IQR 133–460) nmol/L and 3.9 (0.8–6.9) pmol/L, respectively. AI was present in 33 (14%) patients tested, which included 11 patients with severe/critical illness. Among the 33 patients with AI, ACTH was low or inappropriately normal (2.3–12.0 pmol/L) in 30 patients, suggesting hypothalamic–pituitary dysfunction. In the remaining three patients, plasma ACTH was elevated (range 12.3–15.6 pmol/L); however, adrenal imaging was not available for any of them. The clinical and biochemical parameters were comparable between the groups with and without AI (Tables 1 and 2). The median levels of morning serum cortisol, plasma ACTH, and the prevalence of AI did not differ according to the severity of COVID-19 (Supplementary Table 1, see section on supplementary materials given at the end of this article). A higher median cortisol level was noted among the non-survivors (413 nmol/L (158–662)) compared to the survivors (291 nmol/L (130–438)), which showed a trend towards significance (*P* = 0.08), but the median ACTH values were not different between the groups (Supplementary Table 2). In the multivariate analysis, only serum cortisol and T3 levels were independent predictors of mortality (Supplementary Table 3).

A low T3, low TSH, and low FT4 were observed in 70, 29, and 20 subjects, respectively, with either of these abnormalities being noted in 89 patients. While most of these may represent the presence of a nonthyroidal illness syndrome (NTIS), the 20 patients with low FT4 also had a low or normal T3, suggesting the presence of either NTIS or central hypothyroidism. Three patients with severe illness also showed signs of thyrotoxicosis who were negative for TSH receptor antibody. All the biochemical abnormalities recovered at the 12 month follow-up, except in four patients who showed signs of central hypothyroidism. Similarly, the cytokines IL-6 and IL-8 were elevated in 144 and 194 subjects, respectively. Higher levels of cytokines were observed in the group with severe/critical illness (Supplementary Table 1).

### Follow-up assessments at 12 months and onwards

Ninety patients were assessed 12 months after discharge. Long-COVID syndrome was present in 48 (53%) patients. AI, as assessed by APST, was seen in 12 (13%) patients (Table 3). ACTH was low or inappropriately normal (range 0.22–5.06 pmol/L) in all, suggesting a hypothalamic–pituitary origin of the AI. Four patients had a biochemistry suggestive of central hypothyroidism as well; none of them had coexistent AI. Thirty-five of these 90 patients did not have a baseline cortisol value during COVID-19. None of these 35 had AI on APST at 12 months, but two of them had a picture suggestive of central hypothyroidism. Age- and sex-specific DHEAS was low in 21 (23%), and three of them had coexisting AI. The presence of AI was not associated with the severity of acute COVID-19 or with the use of steroids during acute COVID-19 (Table 3). Only two of the 12 patients with AI had received steroids during acute COVID-19 (Table 4). Those with AI had a significantly lower morning cortisol levels during acute illness and a higher prevalence of long-COVID syndrome (Table 3). Of the 12 subjects with AI at 1 year, seven also had evidence of AI at admission for COVID-19 (Table 4). Of the remaining five who had not met the diagnostic criteria for AI during acute COVID, three had morning serum cortisol levels between 100 and 130 nmol/L and two had morning cortisol levels >300 nmol/L (Table 4). Of the 33 patients who had AI during admission, only 14 turned up at 12 months for follow-up. While AI persisted in seven patients, it resolved in the remaining seven.

**Table 3:** Clinical and biochemical parameters in patients with and without adrenal insufficiency at 12 month follow-up.

Parameter	Adrenal insufficiency (*n* = 12)	Normal adrenal function (*n* = 78)	*P*
Age (years)	56 (51–64)	50 (41–60)	0.05
Male sex, *n* (%)	8 (67)	70 (90)	0.03
**During acute COVID-19**			
Severe/critical COVID-19 disease, *n* (%)	5 (42)	15 (19)	0.08
Steroid treatment, *n* (%)	2 (17)	36 (46)	0.05
S. morning cortisol, 0 min (nmol/L)	67.1 (44.8–111.0)	275.2 (90.7–396.3)	0.00
P. ACTH (pmol/L)	6.6 (0.9–8.5)	3.6 (1.2–5.6)	0.11
Adrenal insufficiency during acute COVID-19, *n* (%)	7 (58)	7 (9)	0.02
S. Free thyroxine (pmol/L)	17.0 (11.4–17.6)	16.7 (15.0–19.7)	0.31
S. Triiodothyronine (nmol/L)	1.7 (1.3–1.9)	1.7 (1.3–2.0)	0.88
S. Thyroid-stimulating hormone (mIU/L)	1.9 (1.2–3.1)	1.9 (1.1–3.9)	0.89
Hemoglobin (g/dL)	10.8 (9.4–12.7)	12.1 (10.7–13.5)	0.07
Total leukocyte count (cells/mm^3^)	7980 (5775–16,100)	8100 (5035–11,900)	0.53
ESR (mm/h)	72 (28–94)	50 (6–81)	0.24
S. C-reactive protein (mg/dL)	34.5 (1.0–91.6)	26.5 (8.6–68.9)	0.49
S. Procalcitonin (ng/mL)	0.08 (0.03–0.17)	0.07 (0.03–0.17)	0.72
P. Fibrinogen (mg/dL)	565 (490–683)	504 (424–588)	0.14
P. D-dimer (ng/mL)	0.61 (0.27–2.07)	0.61 (0.26–1.93)	0.59
S. Lactate dehydrogenase (U/L)	356 (245–955)	386 (316–592)	0.98
S. Ferritin (µg/L)	547.5 (90.3–989.5)	461.0 (297.0–765.3)	0.67
P. Interleukin 6 (pg/mL)	33.7 (6.4–157.2)	8.8 (5.0–32.5)	0.17
P. Interleukin 8 (pg/mL)	23.6 (10.2–86.9)	14.8 (9.5–29.9)	0.40
**Parameters at 12-month visit**			
Long-COVID syndrome, *n* (%)	12 (100)	36 (47)	0.00
S. morning cortisol, 0 min (nmol/L)	69 (45–125)	275 (91–396)	0.01
S. peak cortisol (nmol/L)	246 (130–348)	615 (458–754)	0.00
S. DHEAS (µmol/L)	3.82 (0.75–6.56)	2.37 (1.90–3.88)	0.34
S. Free thyroxine (pmol/L)	16.40 (14.51–18.70)	16.28 (14.79–18.26)	0.87
S. Triiodothyronine (nmol/L)	1.3 (1.1–1.7)	1.7 (1.3–2.1)	0.02
S. Thyroid-stimulating hormone (mIU/L)	2.4 (1.9–4.0)	2.1 (1.5–3.4)	0.52

Data are presented as median (interquartile range) and percentage as appropriate.

ACTH, adrenocorticotrophic hormone; COVID-19, coronavirus disease-2019; DHEAS, dehydroepiandrosterone sulfate; ESR, erythrocyte sedimentation rate; P., plasma; S., serum.

**Table 4:** Clinical and biochemical parameters of patients with adrenal insufficiency diagnosed at 12 months follow-up.

Sl no.	Age (y)/Sex	Steroid treatment during acute COVID-19 and cumulative dose	Morning cortisol^a^	ACTH^a^	Morning cortisol^b^	Peak cortisol^b^	Morning cortisol^c^	Peak cortisol^c^
1	59/M	N	24	5.6	195	198	369	564
2	57/F	N^d^	130	6.7	141	296	260	367^e^
3	50/M	Y, 300 mg MP	388	5.6	388	369	^f^	^f^
4	65/M	N^d^	46	0.2	241	228	285	543
5	53/F	N	111	0.2	6	297	629	765
6	52/F	N	71	6.6	249	310	471	679
7	54/M	N	322	7.2	79	184	317	800
8	51/F	N	102	14.5	159	206	^f^	^f^
9	71/M	N	67	0.9	78	211	421	462
10	73/M	Y, 300 mg MP	45	15.6	15	55	430	493
11	62/M	N	64	8.5	49	165	331	472
12	42/M	N	29	3.6	43	288	186	598

Units for cortisol and ACTH are nmol/L and pmol/L respectively.

^a^Assessment during acute COVID-19; ^b^Assessment at 12 months after discharge; ^c^Assessment after 18 months after discharge; ^d^Steroid was not an approved agent at this time for management of severe COVID-19 and hence was not given to these patients, even if they had severe disease; ^e^HPA axis evaluated in ensuing 6 months (24 months after discharge) showed a peak cortisol of 497, suggesting recovery of the HPA axis; ^f^HPA axis could not be evaluated because these patients died (patient #3) or was on high-dose steroids for inflammatory myositis (patient# 8).

ACTH, adrenocortical hormone; COVID-19, coronavirus disease- 2019; MP, methylprednisolone.

All patients diagnosed with AI received steroid replacement therapy. All 12 (100%) patients diagnosed to have AI at 1 year had overlapping symptoms suggestive of long-term COVID and/or AI. Only one patient (patient #2, Table 4) had one episode of adrenal crisis, precipitated by urinary sepsis, till the time that this manuscript was written. We reassessed the HPA axis at 6-month intervals for signs of recovery. Of the 12 patients, one patient had a sudden unexplained death and another required high-dose steroids for inflammatory myositis. The remaining patients showed recovery of the HPA axis at another 6 months of follow-up, except one who on further testing after an additional 6 months showed normalisation of the HPA axis (Table 4, Fig. 1). MRI of the pituitary was done in three patients and did not show any abnormalities in the pituitary or hypothalamus.

**Figure 2:**
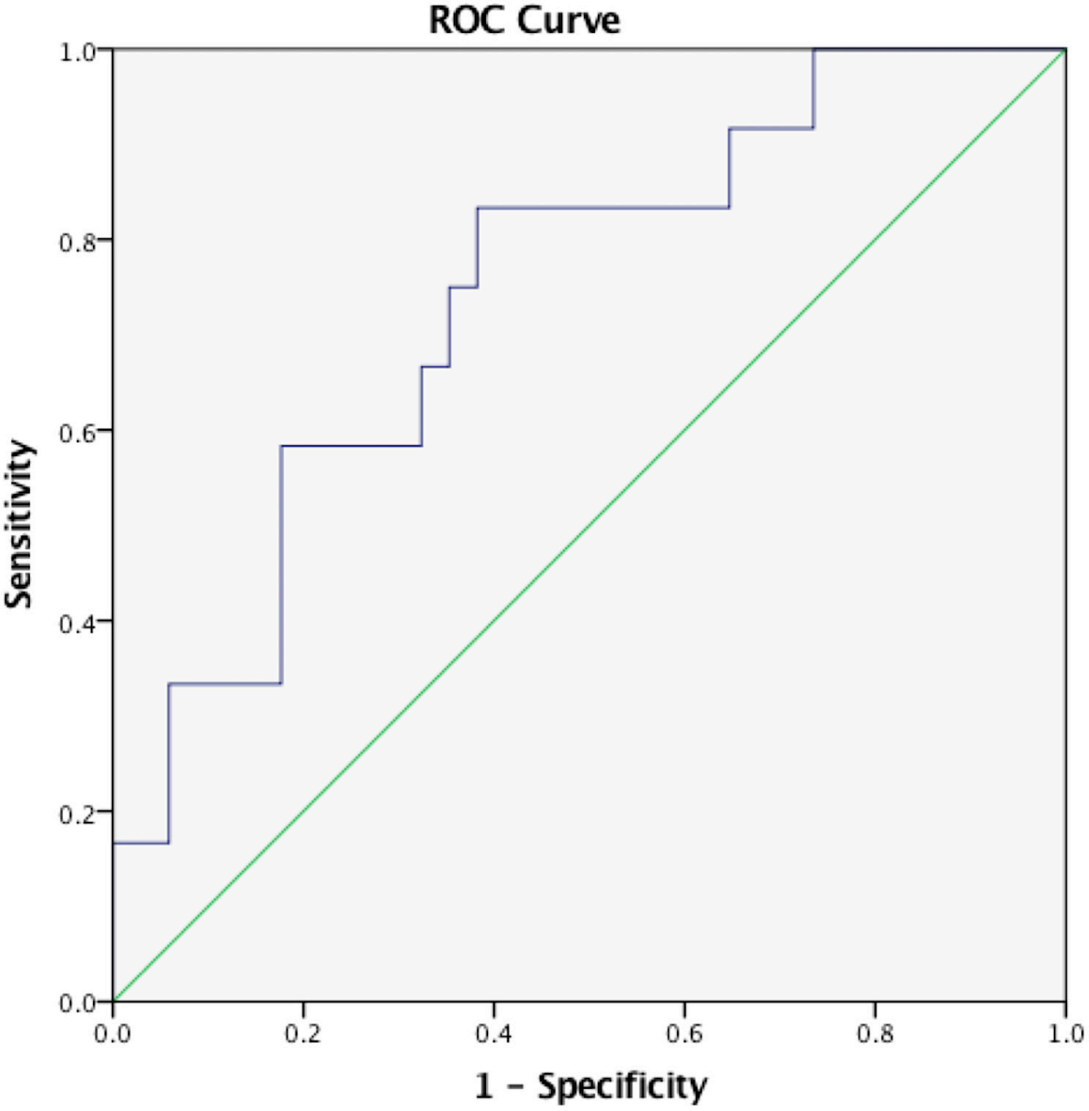
Receiver operating characteristic (ROC) analysis of morning cortisol during acute COVID-19 and presence of adrenal insufficiency at 12 months. The ROC curve for the morning cortisol value during the acute admission in predicting the presence of adrenal insufficiency at 12 months following the discharge. The green line represents the results equivalent to chance alone.

Correlation between various clinical and biochemical parameters are given in the Supplementary Table 4. The morning cortisol measured during acute COVID-19 showed a positive correlation with serum FT4, plasma ACTH, and IL-8 during the acute illness and with morning and peak cortisol at 12 months. It also showed a negative correlation with serum T3 measured during acute COVID-19. In the binary logistic regression model, a morning cortisol of <138 nmol/L during acute COVID-19 predicted the development of AI at 1 year (sensitivity 91% and specificity 62%, Supplementary Table 5). In the ROC analysis of the relationship between morning serum cortisol measured at admission for COVID-19 and presence of AI in follow-up, the area under the curve was 0.74 (95% CI 0.58–0.90; *P* = 0.01, Fig. 2). Baseline morning serum cortisol <388 nmol/L had 100% sensitivity and 27% specificity, while a cortisol <29 nmol/L had 100% specificity and 17% sensitivity in predicting development of AI at 1 year.

## Discussion

In this longitudinal study, we evaluated the HPA axis both during acute COVID-19 and up to 24 months after its recovery. AI was prevalent both during COVID-19 and at 12 months after recovery. AI, both during COVID-19 and in follow-up, was unrelated to the severity of the illness and the use of steroids. The AI at 12 months was of hypothalamic–pituitary origin and recovered in all patients over the ensuing 6–12 months. There was a delayed onset of AI in a subset, in whom the HPA axis was seemingly intact during acute COVID-19. A morning serum cortisol <138 nmol/L during COVID-19 predicted AI at 12 months. The study highlights the importance of assessing AI both during COVID-19 and in follow-up.

There was a considerable degree of dysfunction in the HPA axis during COVID-19. Previous studies have reported varying rates of hypocortisolemia, ranging from 0% to 64% in patients with COVID-19 (3, 4, 5, 6). In our study, we detected a substantial prevalence of AI in a larger cohort while using a stringent cut-off for defining AI. Most subjects had secondary AI, which is consistent with previous reports (4, 5). Plasma ACTH was elevated in three patients with AI, suggesting possible involvement of the adrenal gland. However, adrenal imaging and antibody against 21-hydroxylase were not assessed in these patients. Case reports of primary AI have been reported during acute COVID-19 (24); however, systematic studies assessing the HPA axis have not reported this finding.

Follow-up evaluation at 12 months revealed the presence of secondary AI in 13% of patients. In a study from the United Kingdom, AI was not reported among 70 patients using the SD-SST at least 3 months after recovery from COVID-19 (7). The exclusion criteria in this study prevented the recruitment of patients on glucocorticoid therapy initiated after recovery from COVID-19; thus, it is possible that the study might have missed patients with AI. In contrast, two small-scale studies showed a prevalence of AI in range of 14–16% when tested using a LD-SST after 3 months of recovery (8, 9). Adrenal gland retains sensitivity to stimulation with the ACTH test in the early stages of secondary AI and may result in a false-negative test using the SD-SST (11). Furthermore, the LD-SST is vulnerable to performance errors and may result in false-positive results (12, 13). In our study, we performed the APST, equivalent of SD-SST at a follow-up period of 12 months, a time which should presumably be sufficiently late in the course of secondary AI to eliminate the likelihood of a false-negative result from APST. This is also a period by which the suppressive effects of steroids used during acute COVID-19 would have worn off. Finally, there has been a gradual reduction in the cut-offs used for defining AI with newer assays using the monoclonal antibodies (22). We have used the stringent cut-off for diagnosis of AI, thus reducing the probability of overdiagnosis of AI.

The exact pathophysiology behind AI in COVID-19 is not known. ACE2 receptors, essential for SARS-CoV-2 cellular entry, are expressed in the hypothalamus, pituitary, and adrenals (1, 2). Hence, a direct cytopathic effect of the virus on these glands can result in deficiency of one or more hormones (25, 26, 27, 28). The mRNAs of pituitary hormones were downregulated in patients who died from severe COVID-19; however, biochemical correlations with pituitary hormones were lacking in this study (28). Additionally, SARS-CoV-2 infection may result in endothelial injury, microangiopathy, and thrombosis, which may result in infarction and hypofunction of the adrenal and pituitary glands (26, 27, 29).

Of the 12 patients with AI at 1 year, five did not meet the criteria for AI during COVID-19. In these patients, the AI might have developed after recovery from COVID-19. It is noteworthy that three of these five patients had a morning cortisol <140 nmol/L, and it is difficult to rule out AI in the absence of an appropriate stimulation test, such as insulin tolerance test. The other mechanisms behind a delayed onset of AI could be secondary to reactivation of latent viral infection, post-infectious hypophysitis (30, 31), vaccine medicated (32), or an autoimmunity (33, 34, 35). ‘Molecular mimicry’ between certain epitopes of SARS-CoV-2 and ACTH may result in anti-ACTH antibodies, which further can impair ACTH function (33, 34). Anti-pituitary antibodies have also been demonstrated in patients with COVID-19; whether they play a role in development of hypophysitis and/or ACTH deficiency is not proven (35).

The AI was transient in nature. Of the 14 (out of 33) patients with AI who turned up for the follow-up at 12 months, AI recovered in half. The HPA axis recovered in the following 6–12 months in all the patients diagnosed to have AI at 1 year. The mechanisms behind the transient nature of ACTH deficiency are not known. An infectious, post-infectious, or autoimmune hypophysitis may be plausible explanations. However, we did not find any signal abnormality suggestive of hypophysitis in the limited number of patients who underwent MRI. We also could not measure the anti-pituitary or anti-ACTH antibodies in our patients. In a biologically related SARS-CoV-1 infection, 39% (24/61) of patients had hypocortisolemia at 3 months following recovery from SARS, and hypocortisolemia resolved in all after 1 year (16).

A form of NTIS has been described in patients with COVID-19 similar to our study results (36). Three of our patients with COVID-19 had a reversible form of destructive or inflammatory thyroiditis (37). The thyroid dysfunction recovered in almost all, as noted in several previous studies (38). Four patients had persistent central hypothyroidism, further confirming the hypothalamic–pituitary involvement in patients with COVID-19. Similar to previous studies, most of the patients had an elevated IL-6 and IL-8, which correlated with mortality (39). However, the elevation in cytokines was not related to HPA dysfunction but was related to NTIS.

As most of these patients were previously healthy and had no prior corticosteroid or opiate exposure or known hypothalamic–pituitary disease, there is a strong likelihood that the SARS-CoV-2 was the aetiologic agent responsible for hypocortisolism. This may have widespread implications. Presence of undiagnosed AI during acute COVID-19 may contribute to severe illness and worse outcome. It is established that steroids reduce the morbidity and mortality in patients with severe COVID-19 by reducing the impact of cytokine storm. Glucocorticoids could also have improved the outcome by addressing the underlying COVID-19-induced hypoadrenalism, at least in a subset of patients. Screening of patients with COVID-19 may help in identifying patients with AI, a condition which when treated in a timely manner can prevent further morbidity and mortality.

The study had suffered from a few limitations. Of the 33 patients with AI during acute illness, 11 had severe/critical COVID-19. Cortisol-binding globulin levels are known to get reduced during severe illness (10); hence, it is difficult to predict whether a low total cortisol level truly reflects diagnosis of AI in the absence of measurement of free cortisol. Higher cut-off values of serum cortisol ranging from 280 to 500 nmol/L have been proposed for defining hypocortisolism during acute illness and COVID-19 (40, 41, 42). In the absence of a consensus on the cut-off of serum cortisol value during acute illness, and considering our cohort had a varied severity of illness ranging from asymptomatic to severely ill, we decided to use a single cut-off for homogenously defining AI. Hence, we used the conventional cut-off of 83 nmol/L for this purpose. This might have led to underdiagnosis of AI during COVID-19 in our cohort. We had a large subset of people who did not turn up in follow-up. A subset of patients undergoing evaluation at 12 months for AI also lacked a baseline cortisol measured during COVID-19. Hence, the generalisation of the estimates of our study results should be made with caution. Finally, pituitary and adrenal imaging could not be done for all patients with AI because of COVID-19-related restrictions. The major strengths of the study were careful patient selection and longitudinal evaluation of the HPA axis over a period up to 2 years. The longitudinal evaluation at different time points helped address biases from the use of steroids. We also used much lower cut-offs to define AI, thus reducing the probability of overdiagnosis of AI.

To conclude, AI was present in 14% of patients admitted with COVID-19. We also found that 13% of survivors of COVID-19, tested at 12 months follow-up, had AI. AI developed after recovery from COVID-19 in a subset. The AI was predominantly of hypothalamic–pituitary in origin and resolved in the ensuing 6–12 months. A morning cortisol <138 nmol/L at admission predicted the presence of AI at 12 months. Patients with COVID-19 should be periodically screened for the development of AI and its resolution.

## Supplementary Materials

Supplementary Material

## Declaration of interest

The author(s) declare that there is no conflict of interest that could be perceived as prejudicing the impartiality of the study reported.

## Funding

This work was supported by the Science and Engineering Research Boardhttp://dx.doi.org/10.13039/501100001843, New Delhi, India (Grant number 2020-135-EMP-EXP-20, 2020).

## Data availability

Some or all datasets generated during and/or analysed during the current study are not publicly available but are available from the corresponding author on reasonable request.

## Author contribution statement

SKS and MN was responsible for conceptualisation; SKS and SY were responsible for designing the methodology; SKS was responsible for funding acquisition; SKS, NT, and JCM were responsible for participant recruitment,= and data collection; SKS, JCM, and MN were responsible for clinical data analysis and statistical analyses; SY and SKS were responsible for overall supervision of the project, SKS and JCM wrote the draft; all authors gave critical input, reviewed, and approved the final manuscript.

## Acknowledgements

We thank Mr Manoj Shukla, the Chief Technical Officer and Mr Mukesh Mishra, Medical Laboratory Technician in the department of Endocrinology for running the assays. We acknowledge all the residents and nursing staff who were involved in the care of these patients for their help in sample collection for this project.
