# Supplementary material for: Reversible central adrenal insufficiency in survivors of COVID-19: results from a 24-month longitudinal study
Source: Endocr Connect. 2024 Aug 12;13(9):e240086. doi: 10.1530/EC-24-0086 (PMC11378128; doi:10.1530/EC-24-0086)
Supplement: Supplementary Material [file supplementary_material.pdf]

Reversible central adrenal insufficiency in survivors of COVID19: results from a 24-month longitudinal study

Saroj Kumar Sahoo[1, 2*] (ORCID: 0000-0001-6246-3519), Jayakrishnan C Menon[1] (ORCID: 0000-0002-2658-4505), Nidhi Tripathy[1], Monalisa Nayak[3], Subhash Yadav[1*] (ORCID: 0000-0003-3275-6673)

[1]Department of Endocrinology, Sanjay Gandhi Postgraduate Institute of Medical Sciences, Lucknow, India, [2]Division of Endocrinology, Mid and South Essex NHS Trust, United Kingdom, and [3]Department of Liver Intensive Care Unit, King's College, London, United Kingdom.

* **Corresponding authors**

Saroj Kumar Sahoo and Subhash Yadav

Department of Endocrinology

Sanjay Gandhi Postgraduate Institute of Medical Sciences

Raebareli Road

Lucknow, India 226014

Emails: drsarojsahoo21@gmail.com, subhash70@gmail.com

Supplement table 1: Comparison of parameters according to severity of COVID19

**Parameter**	**Severe disease (n=97)**	**Non-severe disease (n=205)**	**P value**
Age (years)	61 (49.5 - 67)	49 (40 - 61)	0.00
Male sex, n (%)	77 (79.4)	151 (73.7)	0.28
Dyspnoea, n (%)	55 (57)	32 (16)	0.00
Symptom duration (day)	7 (4 - 10)	4 (2 - 7)	0.00
Diabetes mellitus, n (%)	56 (57.7)	82 (40)	0.01
Hypertension, n (%)	56 (57.7)	90 (43.9)	0.03
Pneumonia, n (%)	90 (94)	91 (46)	0.00
CT severity score (n = 98)	15 (11 - 19)	6 (3 - 10)	0.00
Respiratory failure, n (%)	96 (99)	0	0.00
Non-invasive ventilation, n (%)	26 (27)	0	0.00
Mechanical ventilation, n (%)	16 (17)	0	0.00
ARDS (n=283) , n (%)	43 (46)	0	0.00
Steroid use, n(%)	56 (58)	52 (26)	0.00
Remdesivir use, n (%)	64 (66)	80 (39)	0.00
Death, n (%)	23 (24)	3 (1.5)	0.00
S. morning cortisol (nmol/L)	334 (147- 512)	274 (122- 438)	0.17
P. ACTH (pmol/L)	3.6 (0.5 – 6.9)	4.0 (1.2 – 6.9)	0.25
S. free thyroxine (pmol/L)	17.0 (14.4-18.9)	17.4 (15.2-20.0)	0.11
S. triiodothyronine (nmol/L)	1.3 (1.1-1.7)	1.7 (1.4-2.0)	0.00
S. TSH (mIU/L)	1.4 (0.8-2.8)	2.0 (1.0-3.2)	0.03
Hemoglobin (g/L)	11 (9.6 - 12.4)	11.9 (10.2 - 13.3)	0.01
Leukocyte count (cells/mm3)	13400 (7820 - 18200)	7800 (5700 - 10900)	0.00
ESR (mm/hr)	79.5 (52 - 97)	36.5 (6.8 - 74)	0.00
S. C-reactive protein (mg/dL)	95.3 (44 – 173.8)	17.0 (5.0 – 56.1)	0.00
S. Procalcitonin (ng/mL)	0.15 (0.08 - 0.67)	0.04 (0.02 - 0.15)	0.00
P. Fibrinogen (mg/dL)	575.0 (498.3 – 661.3)	492.6 (394.0 – 569.8)	0.00
P. D-dimer (ng/mL)	0.86 (0.24 - 3.68)	0.47 (0.19 - 1.58)	0.59
S. lactate dehydrogenase (U/L)	605 (372 - 930)	367 (271 – 526.8)	0.00
S. Ferritin (ug/L)	855 (401 – 1921)	338 (158 – 652.5)	0.00
P. interleukin 6 (pg/mL)	21.8 (5 - 85.8)	7.0 (5 - 22.8)	0.00
P. interleukin 8 (pg/mL)	33.2 (9.9 - 81.3)	13.3 (7.4 - 28.6)	0.00
AI during COVID19 (n=231), n (%)	11 (13.4)	22 (14.3)	0.78
AI at follow up (n = 90)	5 (25%)	7 (10%)	0.08

Data are expressed as median (IQR) and percentage.

ACTH: Adrenocorticotrophic hormone, AI: Adrenal insufficiency, ARDS: Acute respiratory distress syndrome, COVID19: Corona-virus disease 19, CT: Computer tomography, ESR: Erythrocyte sedimentation rate, TSH: Thyroid stimulating hormone.

Supplement table 2: Comparison of parameters among survivors and non-survivors in patients with COVID19

**Parameter**	**Non-survivors (n=30)**	**Survivors (n=272)**	**P value**
Age (years)	61 (52-71)	53 (42-64)	0.01
Male sex, n (%)	23 (77)	205 (76)	1.00
Dyspnoea, n (%)	18 (60)	69 (25)	0.00
Symptom duration (day)	5 (2-7)	5 (3-8)	0.89
Diabetes mellitus, n (%)	19 (63)	119 (44)	0.14
Hypertension, n (%)	16 (53)	130 (48)	0.57
Pneumonia, n (%)	29 (97)	153 (58)	0.00
CT severity score	19 (13-23)	10 (5-14)	0.00
Respiratory failure, n (%)	26 (97)	71 (28)	0.00
Non-invasive ventilation, n (%)	19 (63)	7 (3)	0.00
Mechanical ventilation, n (%)	16 (53)	0	0.00
ARDS (n=283), n (%)	19 (68)	23 (9)	0.00
Steroid use, n(%)	12 (40)	96 (35)	0.69
S. morning cortisol (nmol/L)	413 (158-662)	291 (130-438)	0.08
P. ACTH (pmol/L)	4.1 (1.2-7.8)	4.0 (0.7-6.9)	0.59
S. free thyroxine (pmol/L)	17.0 (12.3-19.5)	17.2 (15.1-20.0)	0.33
S. triiodothyronine (nmol/L)	1.1 (0.9-1.4)	1.6 (1.3-2.0)	0.00
S. TSH (mIU/L)	1.0 (0.4-1.5)	2.0 (1.0-3.2)	0.00
ESR (mm/hr)	81 (52-105)	54 (14-81)	0.00
S. C-reactive protein (mg/dL)	185.5 (82-253.0)	26.0 (8.0-73.5)	0.00
S. Procalcitonin (ng/mL)	1.33 (0.27-5.82)	0.06 (0.03-0.18)	0.00
P. Fibrinogen (mg/dL)	676 (585-712)	514 (415-588)	0.00
P. D-dimer (ng/mL)	3.68 (1.42-8.02)	0.51 (0.19-1.35)	0.00
S. lactate dehydrogenase (U/L)	711 (398-1722)	393 (287-592)	0.00
S. Ferritin (ug/L)	1389 (476-4879)	397 (202-863)	0.00
P. interleukin 6 (pg/mL)	40 (5-265)	8.3 (5-31)	0.00
P. interleukin 8 (pg/mL)	72 (11-268)	15 (8-39)	0.00

Data are expressed as median (IQR) and percentage.

ACTH: Adrenocorticotrophic hormone, AI: Adrenal insufficiency, ARDS: Acute respiratory distress syndrome, COVID19: Corona-virus disease 19, CT: Computer tomography, ESR: Erythrocyte sedimentation rate, TSH: Thyroid stimulating hormone.

Supplement Table 3: Predictor of mortality in the cohort with COVID19

**Parameter**	**B**	**SE**	**Exp B**	**95% CI**	**p**
Age	-0.001	0.024	0.999	0.953–1.048	0.98
Baseline cortisol	0.003	0.001	1.003	1.001–1.005	0.00
TSH	-0.216	0.166	0.806	0.582–1.115	0.19
Triiodothyronine	-3.564	1.132	0.028	0.003–0.260	0.00
Respiratory failure	16.188	40192	10722243	0.000	1.00
C-reactive protein	0.000	0.001	1.000	0.999–1.001	0.63
Ferritin	0.000	0.000	1.000	1.000–1.000	0.06
D-Dimer	-0.003	0.026	0.997	0.946–1.050	0.90
Procalcitonin	0.002	0.007	1.002	0.988–1.016	0.77
Interleukin 6	0.000	0.001	1.000	0.998–1.002	0.67
Interleukin 8	0.001	0.001	1.001	0.998–1.004	0.42
Constant	-15.165	40192.876	0.000		

CI: Confidence interval, COVID19: Corona-virus disease 19, SE: standard error, TSH: Thyroid stimulating hormone.

Supplement table 4: Correlation among various biochemical parameters

	Baseline cortisol 0	Age	TSH 0	FT4 0	T3 0	ACTH 0	Baseline cortisol 1	Peak cortisol 1	TSH 1	FT4 1	T3 1	DHEAS 1	SBP	DBP	CRP	Ferritin	D-Dimer	Fibrinogen	IL6	IL8
Baseline cortisol 0	1	.109	.023	.181**	-.005*	.176**	.614**	.718*	-.126	.059	.103	-.270	-.066	-.099	-.008	.081	-.034	-.008	.082	.168*
Age	.109	1	-.050	.005	-.163**	-.057	-.033	-.692**	-.235*	-.115	.026	-.034	.072	-.103	.074	.026	-.073	.152*	.137*	.100
TSH 0	.023	-.050	1	-.051	.044	-.057	-.049	.191	.217	.021	-.021	-.116	.050	.042	-.056	-.073	-.024	-.129	-.012	.315**
FT4 0	.181**	.005	-.051	1	-.016	.082	-.191	.414	-.040	-.119	.143	.133	.039	.022	-.006	-.020	.002	-.050	-.026	-.113
T3 0	-.005*	-.163**	.044	-.016	1	.023	.019	-.095	.156	-.167	-.165	-.048	-.010	.092	-.114	-.151*	-.052	-.140	-.157*	-.142*
ACTH 0	.176**	-.057	-.057	.082	.023	1	.000	-.278	-.067	-.010	-.076	-.238	.112	.024	-.015	-.028	-.012	-.049	-.003	.017
Baseline cortisol 1	.614**	-.033	-.049	-.191	.019	.000	1	.164	-.047	.082	.067	-.073	.007	-.024	.033	-.136	.056	-.205	.056	.076
Peak cortisol 1	.718*	-.692**	.191	.414	-.095	-.278	.164	1	.095	.066*	.353	-.300	-.313	-.034	-.191	-.148	.173	-.012	-.215	-.108
TSH 1	-.126	-.235*	.217	-.040	.156	-.067	-.047	.095	1	-.001	-.115	.025	-.001	.111	-.041	-.057	-.039	-.267*	-.110	-.084
FT4 1	.059	-.115	.021	-.119	-.167	-.010	.082	.066*	-.001	1	-.069	.046	.276**	.217*	-.062	-.048	-.050	.017	.081	.098
T3 1	.103	.026	-.021	.143	-.165	-.076	.067	.353	-.115	-.069	1	.104	.024	.077	-.085	-.012	.227*	.009	.125	.138
DHEAS 1	-.270	-.034	-.116	.133	-.048	-.238	-.073	-.300	.025	.046	.104	1	-.065	-.001	.068	-.061	.096	-.008	-.057	.079
SBP	-.066	.072	.050	.039	-.010	.112	.007	-.313	-.001	.276**	.024	-.065	1	.702**	-.056	.033	.039	.045	.022	-.001
DBP	-.099	-.103	.042	.022	.092	.024	-.024	-.034	.111	.217*	.077	-.001	.702**	1	-.042	-.026	.013	-.014	.015	.016
CRP	-.008	.074	-.056	-.006	-.114	-.015	.033	-.191	-.041	-.062	-.085	.068	-.056	-.042	1	.016	-.004	.046	.006	.004
Ferritin	.081	.026	-.073	-.020	-.151*	-.028	-.136	-.148	-.057	-.048	-.012	-.061	.033	-.026	.016	1	-.015	.150*	.041	.001
D-Dimer	-.034	-.073	-.024	.002	-.052	-.012	.056	.173	-.039	-.050	.227*	.096	.039	.013	-.004	-.015	1	-.059	.002	-.019
Fibrinogen	-.008	.152*	-.129	-.050	-.140	-.049	-.205	-.012	-.267*	.017	.009	-.008	.045	-.014	.046	.150*	-.059	1	.264**	.119
IL6	.082	.137*	-.012	-.026	-.157*	-.003	.056	-.215	-.110	.081	.125	-.057	.022	.015	.006	.041	.002	.264**	1	.353**
IL8	.168*	.100	.315**	-.113	-.142*	.017	.076	-.108	-.084	.098	.138	.079	-.001	.016	.004	.001	-.019	.119	.353**	1

*. Correlation is significant at the 0.05 level (2-tailed).
**. Correlation is significant at the 0.01 level (2-tailed).
0: evaluation during COVID19, 1: evaluation at 12 months of follow-up

ACTH: Adrenocorticotrophic hormone, CRP: C-reactive protein, CT: Computer tomography, DBP: Diastolic blood pressure, DHEAS: Dehydroepiandrosterone sulfate, ESR: Erythrocyte sedimentation rate, FDP: Fibrin-degradation product, FT4: Free thyroxine, IL6: Interleukin 6, IL8: Interleukin 8, SBP: Systolic blood pressure, T3: Triiodothyronine, TSH: Thyroid stimulating hormone

Supplement Table 5: Baseline parameters predicting adrenal insufficiency at 12-month follow-up.

**Predictors**	**B**	**SE**	**Exp B**	**95% CI**	**P value**
Age	0.05	0.03	1.05	0.98–1.13	0.14
Male gender	-1.12	0.95	0.33	0.05–2.10	0.24
Steroid treatment during acute admission	-1.07	0.98	0.34	0.05–2.33	0.27
Basal cortisol during acute admission	-0.007	0.003	0.99	0.99–1.00	0.04
Constant	-1.48	1.99	0.23		0.46

CI: Confidence interval, SE: Standard error
